# The expression profiles of signature genes from CD103^+^LAG3^+^ tumour-infiltrating lymphocyte subsets predict breast cancer survival

**DOI:** 10.1186/s12916-023-02960-1

**Published:** 2023-07-24

**Authors:** Zi-An Xia, Can Lu, Can Pan, Jia Li, Jun Li, Yitao Mao, Lunquan Sun, Jiang He

**Affiliations:** 1 Department of Integrated Traditional Chinese and Western Medicine, Xiangya Hospital, Central South University, Changsha, 410008 China; 2 National Clinical Research Center for Geriatric Disorders, XiangyaHospital, Central South University, Changsha, 410008 China; 3 Department of Pathology, Xiangya Hospital, Central South University, Changsha, 410078 China; 4 School of Clinical Medicine, Hunan University of Traditional Chinese Medicine, Changsha, 410208 China; 5 Department of Emergency, Xiangya Hospital, Central South University, Changsha, 410008 China; 6 Department of Nuclear Medicine, Peking University Shenzhen Hospital, Guangdong, 518036 China; 7 Department of Radiology, Xiangya Hospital, Central South University, Changsha, 410078 China; 8 Department of Oncology, Xiangya Cancer Center, XiangyaHospital, Central South University, Changsha, 410008 China; 9 Key Laboratory of Molecular Radiation Oncology Hunan Province, Changsha, 410008 China; 10 Hunan International Science and Technology Collaboration Base of Precision Medicine for Cancer, Changsha, 410008 China; 11 Center for Molecular Imaging of Central, South University, Xiangya Hospital, Changsha, 410008 China

**Keywords:** Large-scale data analysis, Tumour-infiltrating lymphocytes, CD103, LAG3, Immunotherapy, Chemotherapy

## Abstract

**Background:**

Tumour-infiltrating lymphocytes (TILs), including T and B cells, have been demonstrated to be associated with tumour progression. However, the different subpopulations of TILs and their roles in breast cancer remain poorly understood. Large-scale analysis using multiomics data could uncover potential mechanisms and provide promising biomarkers for predicting immunotherapy response.

**Methods:**

Single-cell transcriptome data for breast cancer samples were analysed to identify unique TIL subsets. Based on the expression profiles of marker genes in these subsets, a TIL-related prognostic model was developed by univariate and multivariate Cox analyses and LASSO regression for the TCGA training cohort containing 1089 breast cancer patients. Multiplex immunohistochemistry was used to confirm the presence of TIL subsets in breast cancer samples. The model was validated with a large-scale transcriptomic dataset for 3619 breast cancer patients, including the METABRIC cohort, six chemotherapy transcriptomic cohorts, and two immunotherapy transcriptomic cohorts.

**Results:**

We identified two TIL subsets with high expression of CD103 and LAG3 (CD103^+^LAG3^+^), including a CD8^+^ T-cell subset and a B-cell subset. Based on the expression profiles of marker genes in these two subpopulations, we further developed a CD103^+^LAG3^+^ TIL-related prognostic model (CLTRP) based on CXCL13 and BIRC3 genes for predicting the prognosis of breast cancer patients. CLTRP-low patients had a better prognosis than CLTRP-high patients. The comprehensive results showed that a low CLTRP score was associated with a high TP53 mutation rate, high infiltration of CD8 T cells, helper T cells, and CD4 T cells, high sensitivity to chemotherapeutic drugs, and a good response to immunotherapy. In contrast, a high CLTRP score was correlated with a low TP53 mutation rate, high infiltration of M0 and M2 macrophages, low sensitivity to chemotherapeutic drugs, and a poor response to immunotherapy.

**Conclusions:**

Our present study showed that the CLTRP score is a promising biomarker for distinguishing prognosis, drug sensitivity, molecular and immune characteristics, and immunotherapy outcomes in breast cancer patients. The CLTRP could serve as a valuable tool for clinical decision making regarding immunotherapy.

**Supplementary Information:**

The online version contains supplementary material available at 10.1186/s12916-023-02960-1.

## Background

Breast cancer is the most common cancer type worldwide. In 2020, there were approximately 2.3 million newly diagnosed breast cancer cases and 680,000 related deaths worldwide [1]. Currently, surgical resection with adjuvant chemoradiotherapy or hormone therapy are the gold standard for treating breast cancer patients [2]. Immune checkpoint therapy (ICT), as a new therapeutic approach, has been used to treat breast cancer patients [3–5], but most patients do not respond to ICT [6], and there are no available biomarkers to predict the response. Recent studies have shown that breast cancer is an immunogenic cancer type and contains large quantities of tumour-infiltrating lymphocytes (TILs) [7, 8], suggesting that TILs may be associated with the immunotherapy outcomes of breast cancer.

TILs are composed of T cells and B cells and have been demonstrated to be associated with the development of breast cancer [9]. CD103 is expressed on subsets of CD8^+^ T cells and is essential for antitumour cytotoxic T-cell activity because it triggers lytic granule polarization and release at contact sites [10]. In addition, CD103 binds to its ligand, E-cadherin, on epithelial tumour cells, leading to the retention of antigen-specific lymphocytes within epithelial tumours [11]. Thus, CD103 is considered a crucial marker of tissue-resident memory T (TRM) cells. Patients with advanced-stage breast cancers with high levels of TRM cells have better response rates to anti-PD-1 antibodies than those with low levels of TRM cells [7]. Lymphocyte activation gene 3 (LAG3), an immune checkpoint molecule, is expressed on multiple cell types, including CD4^+^ and CD8^+^ T cells [12]. Persistent antigen stimulation in cancer leads to upregulation of LAG3 expression, promoting T-cell exhaustion [12, 13]. Thus, an increasing number of studies have used LAG3 to mark exhausted T cells [14–19]. Single-cell RNA sequencing (scRNA-seq) is a powerful technique for dissecting the heterogeneity of solid tumours [20], which will pave the way for individualized treatment. ScRNA-seq analysis of the tumour microenvironment contributes to identifying immune cell subsets associated with prognosis and understanding their molecular characteristics, which provides an effective way to predict the immunotherapy response and prognosis of cancer patients. Therefore, identification of potential prognostic markers associated with TIL subpopulations based on integrated analysis of scRNA and bulk RNA sequencing and machine learning algorithms might provide effective ICT outcome prediction and therapeutic indicators for breast cancer patients.

In our present study, two CD103^+^LAG3^+^ TIL subsets that were associated with antitumour immunity were identified by scRNA-seq analysis. Based on the expression profiles of marker genes in these two subsets, we constructed a CD103^+^LAG3^+^ TIL-related risk score prognostic model (CLTRP) by performing least absolute shrinkage and selection operator (LASSO) regression and Cox analysis. We used the model to predict overall survival and explored the molecular characteristics, immune infiltration, and chemotherapeutic sensitivity of different CLTRP subgroups. Furthermore, the ability of this risk score prognostic model to predict patient response to chemotherapy and immunotherapy was assessed.

## Methods

### Data sources and study design

**Fig. 1:**
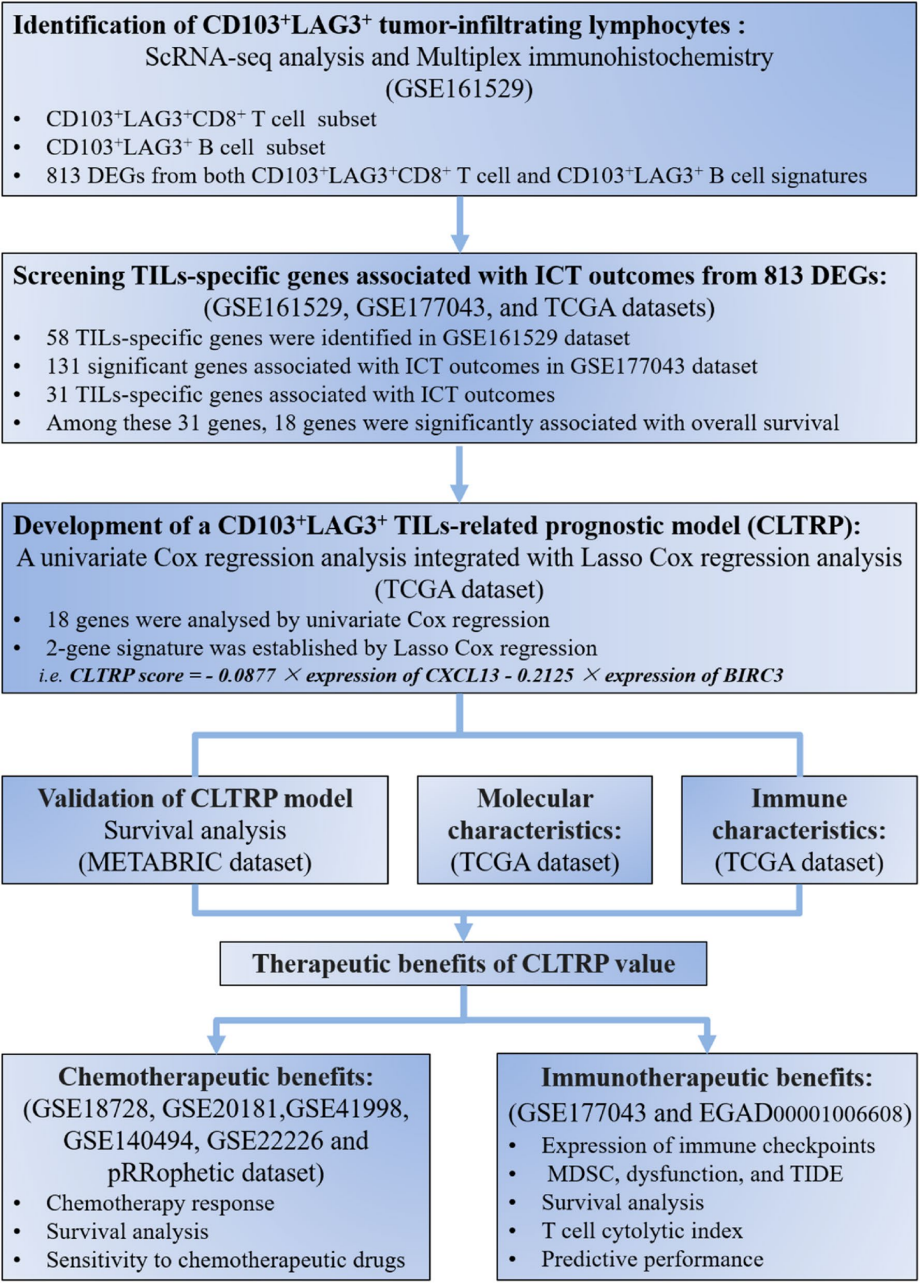
Workflow of the present study

Open breast cancer gene expression datasets with complete prognostic and clinicopathological information annotation were downloaded from The Cancer Genome Atlas (TCGA) (*n* = 1089) and Molecular Taxonomy of Breast Cancer International Consortium (METABRIC) (*n* = 1904) databases. Single-cell RNA-sequencing data (accession number GEO: GSE161529 [21]) of breast cancer samples from the initial publication were analysed to identify CD103^+^LAG3^+^ TILs. The GSE18728 [22], GSE20181 [23], GSE41998 [24], GSE140494 [25], GSE22226 [26], and pRRophetic [27] datasets were used to analyse the chemotherapy response of CLTRP subgroups. Two cohorts of breast cancer patients treated with PD-1/PD-L1 antibodies (GSE177043 [28] and EGAD00001006608 [29]) were obtained to evaluate the predictive performance of CLTRP. Gene mutation information was obtained from the cBioPortal database. The workflow of the present study is shown in Fig. 1.

### Multiplex immunohistochemistry

We performed multiplex immunohistochemistry using the OPAL serial immunostaining protocol as previously described [30]. Briefly, FFPE sections were incubated for 30 min at room temperature with rabbit anti-human CD8 (ZSGB-BIO, catalogue no. ZA-0508), anti-human integrin alpha E (CD103) (ZSGB-BIO, catalogue no. ZA-0667), anti-human CD79A (ZSGB-BIO, catalogue no. ZA-0293), and anti-human LAG3 (1:200, Sigma, catalogue no. HPA013967). The sections were washed three times in PBS buffer. After the addition of secondary horseradish peroxidase–conjugated antibody provided by PerkinElmer, the sections were incubated for 10 min at room temperature. Then, the sections were incubated for 10 min at room temperature with TSA Plus working solution as specified by the manufacturer. Multispectral imaging was performed using a Vectra 3.0 instrument (PerkinElmer) at 20 × magnification.

### Quality control and cell type recognition

An analysis of scRNA-seq data from breast cancer samples (GSE161529) was performed using the Seurat R package (version 4.0.4) [31]. According to previously reported quality control criteria [21], single cells with < 200 genes or UMI count < 1000 or the percent of mitochondrial genes over 20% of total expressed genes were screened as low-quality cells and eliminated. Thereafter, the major cell types were recognized based on previously reported markers [32]. After preprocessing, normalization, and batch correction, T cells and B cells were reclustered separately to identify CD103^+^LAG3^+^ TILs. The signature genes were selected according to the criteria of absolute value of log2FC > 0.5 and *p*_value < 0.05.

### Development of a CD103^+^LAG3^+^ TIL-related prognostic model

Firstly, we obtained differentially expressed genes (DEGs) with an absolute value of log2FC > 0.5 from CD103^+^LAG3^+^TIL subpopulations when compared with other T or B-cell subpopulations. Afterward, we screened TIL-specific genes based on the expression of DEGs in non-lymphocyte populations, and the GSE177043 dataset was used to identify TIL-specific genes significantly associated with ICT outcomes. These TIL-specific genes associated with ICT outcomes were subjected to univariate Cox analysis. Subsequently, least absolute shrinkage and selection operator (LASSO) Cox repression [33] was used to determine the most powerful prognostic genes. Finally, two genes (CXCL13 and BIRC3) and their correlative coefficients were obtained to construct the CD103^+^LAG3^+^ tumour-infiltrating lymphocyte-related gene prognostic model (CLTRP). Based on the median CLTRP score, the 1089 breast cancer samples from the TCGA dataset were divided into low and high CLTRP score subgroups. Then, survival analysis was performed for the two groups using the survival R package (version 3.2.13), and the results are shown using Kaplan‒Meier plots. Finally, receiver operating characteristic (ROC) curve analysis was performed using the survivalROC R package to obtain the area under the curve (AUC) value and evaluate the predictive performance of the signature. Moreover, the METABRIC database was used to validate the predictive ability of the CLTRP model.

### Comprehensive analysis of immune characteristics in different CLTRP subgroups

To identify the immune characteristics of breast cancer patients in the TCGA cohort, their expression data were imported into the CIBERSORT function, and analysis with 1000 resamples was performed to estimate the relative proportion of 22 types of immune cells. Then, we compared the relative proportions of 22 types of immune cells between the two CLTRP subtypes, and the results are presented in a landscape map. The stromal score, immune score, ESTIMATE score, and tumour purity were analysed using the estimate R package. Tumour Immune Dysfunction and Exclusion (TIDE) analysis (http://tide.dfci.harvard.edu/) was performed to predict immunotherapeutic response. The T-cell cytolytic index was evaluated by the expression levels of GZMA and PRF1 as previously described [34].

### Functional and pathway enrichment analysis

DEGs between the high- and low-CLTRP score groups were identified by the limma R package. To further explore the potential functions of DEGs, gene ontology (GO) and Kyoto Encyclopedia of Genes and Genomes (KEGG) enrichment analyses were performed on the DEGs using the clusterProfiler R package (version 4.0.5) [35]. Moreover, to determine the different pathways for analysing the differences in biological function between high- and low-score groups, the gene set variation analysis (GSVA) package (version 1.40.1) was applied for calculating GSVA scores of hallmark gene sets from the Molecular Signatures Database (MSigDB) [36]. *P* values less than 0.05 were considered to indicate significant differences.

### Mutation and drug sensitivity analysis

To reveal relevant genetic alterations, the somatic mutations of CLTRP subgroups were analysed. The mutation annotation format (MAF) from the TCGA database was generated using the “maftools” R package. To investigate the differences in the therapeutic effects of chemotherapeutic drugs in breast cancer patients between the two subgroups, we calculated the half-maximal inhibitory concentration (IC50) values of chemotherapeutic drugs commonly used to treat breast cancer using the “pRRophetic” package.

### Identification of cohorts with immune checkpoint blockade treatment

Two cohorts of breast cancer patients (GSE177043 and EGAD00001006608) treated with PD-1/PD-L1 antibodies had relatively complete clinical information, including follow-up information and immunotherapy effect information. Samples with incomplete clinical information were eliminated from the follow-up analysis. We used the surv_cutpoint function in the survminer R package to calculate the optimal cut-off value for survival analysis. The AUC values were calculated to evaluate the predictive performance of immunotherapy using the pROC package.

### Statistical analysis

Statistical analyses were performed using R software (version 4.1.1). Additional file 1: Table S1 shows the corresponding R codes. All *p* values less than 0.05 (*p* < 0.05) were considered statistically significant.

## Results

### Identification of the CD8^+^CD103^+^LAG3^+^ T-cell subset and CD103^+^LAG3^+^ B-cell subset in breast cancer by scRNA-seq

To identify potential TIL subsets that were associated with antitumour immunity, we reanalyzed scRNA-seq data derived from breast cancer samples [21]. First, these original single cells were divided into immune cells (CD45^+^) and nonimmune cells (CD45^−^) and were visualized by t-distributed stochastic neighbour embedding (t-SNE) (Additional file 2: Fig. S1A). The immune cells were reclustered separately (Additional file 2: Fig. S1B). Expression of genes defining T and B cells is shown for humans in Additional file 2: Fig. S1C, D.

**Fig. 2:**
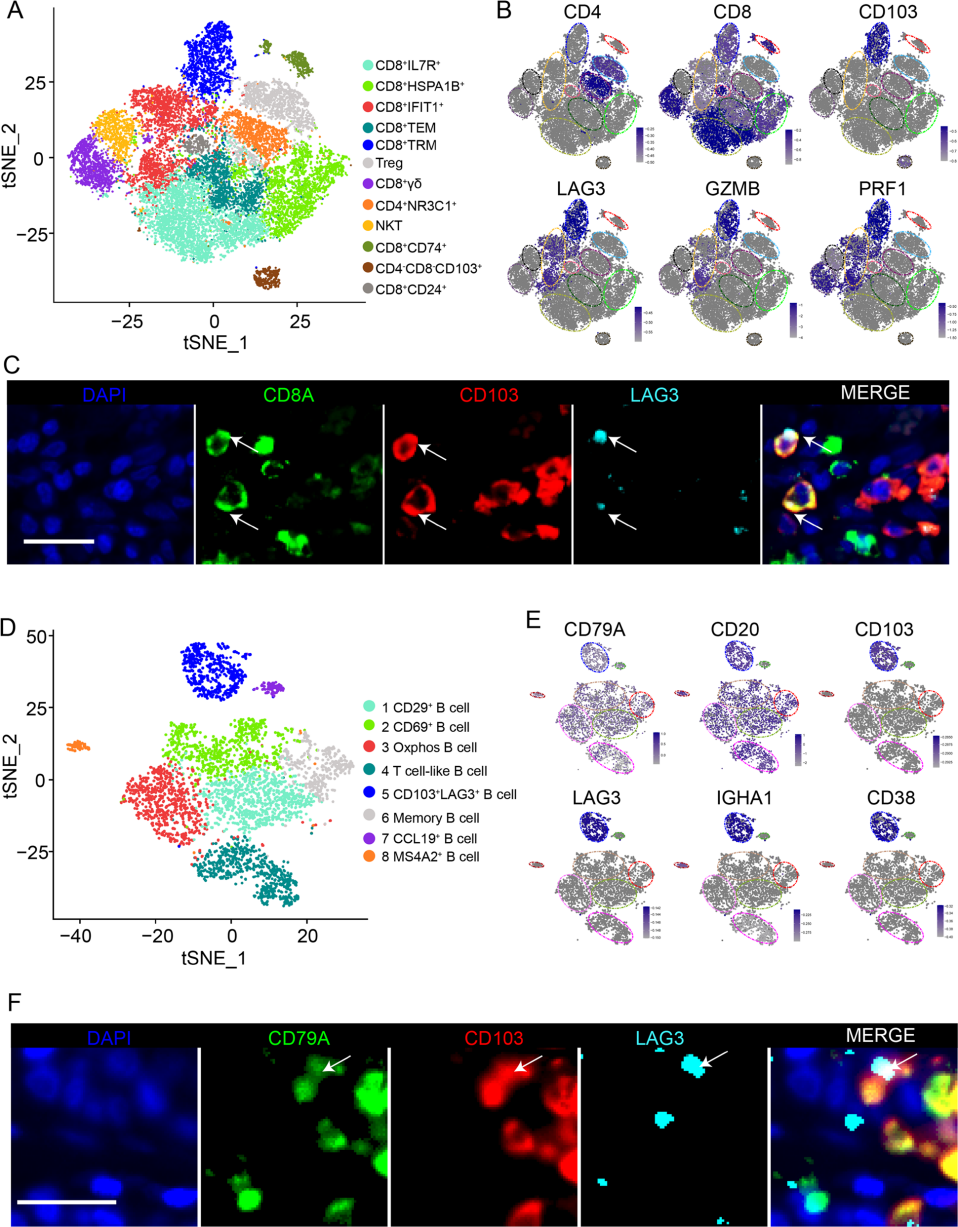
Classification of T and B cells in breast cancer into different subpopulations. **A** The t-SNE plot of T cells. **B** Feature plots showing the expression of key genes in T cells. The colour scale represents the SAVER imputed gene expression value (log10 scale) for each cell. **C** Representative images of breast cancer sections stained by fluorescent multiplex immunohistochemistry showing the expression of CD103, LAG3, and CD8 in T cells. **D** The t-SNE plot of B cells. **E** The t-SNE plots showing the average expression of BRM marker genes. **F** Representative images of breast cancer sections stained by fluorescent multiplex immunohistochemistry showing the expression of CD103, LAG3, and CD79A in B cells. Scale bar, 50 μm

T-cell clusters were observed using t-SNE and showed high heterogeneity among patients (Fig. 2A and Additional file 2: Fig. S2A). A total of 12 unique clusters were identified based on their gene expression profiles (Additional file 2: Fig. S2B). Additional file 1: Table S2 presents significant marker genes of each T-cell subset. The subsets included eight distinct CD8^+^ T-cell subsets, one CD4^−^CD8^−^ T-cell subset, one NKT subset, and two CD4^+^ T-cell subsets (Fig. 2A).

When focusing on the different CD8^+^ clusters, we noted that one cluster had an expression profile suggestive of a TRM cell phenotype. This CD8^+^ TRM-like subset expressed CD103 at high levels and SELL and KLF2 at low levels (Fig. 2B and Additional file 2: Fig. S2C), similar to TRM cells described in humans. Interestingly, we also observed that this subset had high expression of the immune checkpoint molecule LAG3 (Fig. 2B), suggesting that this subset is in an exhausted state. Therefore, this CD8^+^ TRM-like subset was further named the CD8^+^CD103^+^LAG3^+^ T cell cluster. In addition to CD103 and LAG3, this subset exhibited upregulation of effector genes, including granzyme B (GZMB) and perforin (PRF1) (Fig. 2B). Multiplex immunohistochemistry showed the presence of CD8^+^CD103^+^LAD3^+^ T cells in breast cancer (Fig. 2C).

To explore the heterogeneity of B cells, 4180 B cells were individually reclustered (Fig. 2D). The top five markers of the main cell lineages were visualized in a bubble chart (Additional file 2: Fig. S2D). Additional file 1: Table S3 shows the significant marker genes of each B-cell subset. We noted that a B-cell subset specifically expressed CD103 and LAG3 (Fig. 2E). Interestingly, we observed that this cluster also highly expressed effector B-cell marker genes, including CD38 and CXCR4, and memory B-cell marker genes, such as CD20 (MS4A1), IgG, and IgA (Fig. 2E and Additional file 2: Fig. S2E). Thus, this cluster was defined as the CD103^+^LAG3^+^ B-cell subset. These results suggest that the CD103^+^LAG3^+^ B-cell subset may have an antitumour function in breast cancer. Multiplex immunohistochemistry showed the presence of CD103^+^LAG3^+^ B cells in breast cancer (Fig. 2F).

### Identification of TIL-specific genes associated with ICT outcomes

**Fig. 3:**
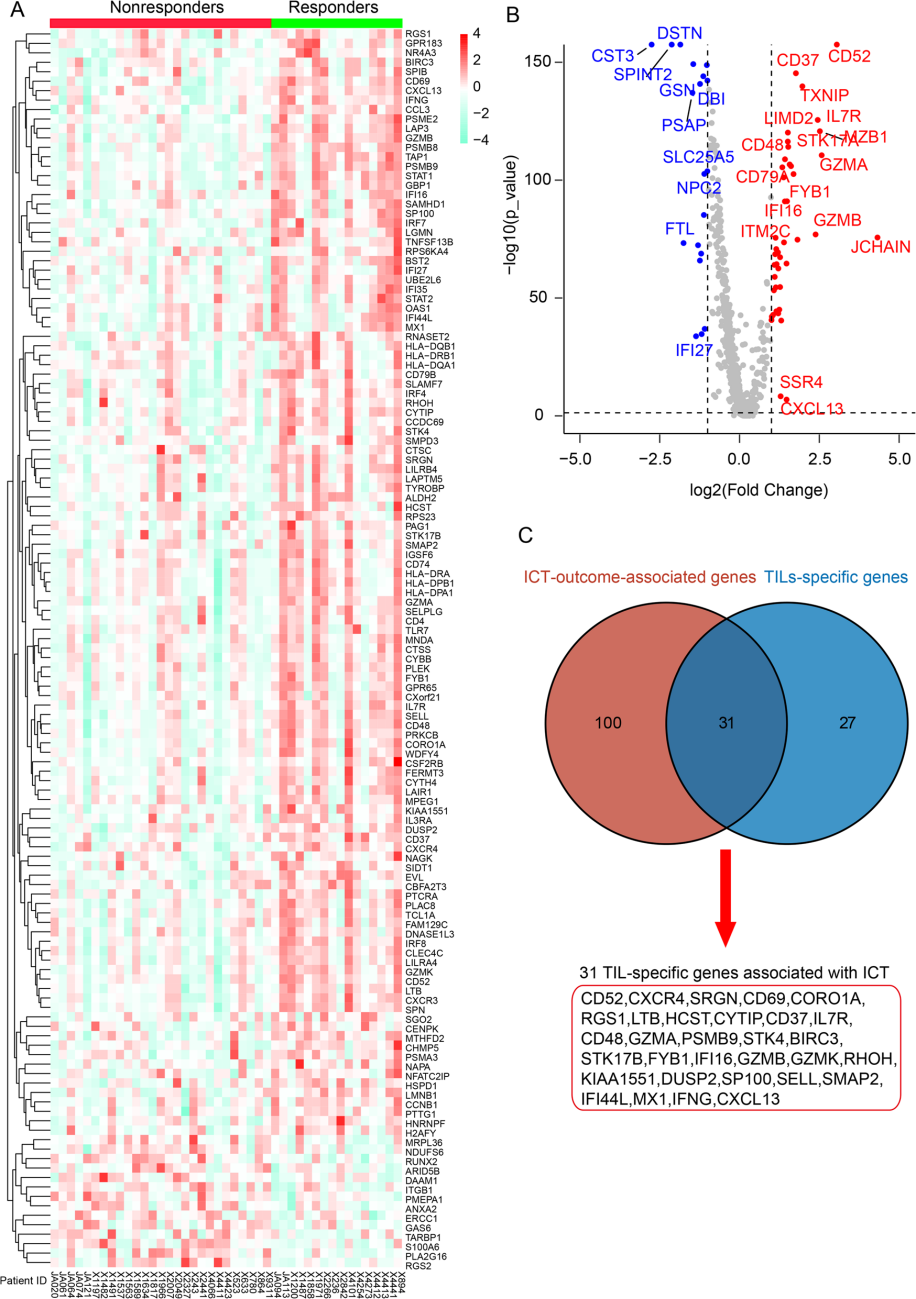
Identification of genes both associated with immunotherapy outcomes and uniquely expressed in TILs. **A** Heatmap exhibiting the expression of ICT-outcome-associated genes in breast cancer cohort receiving anti-PD1 therapy. **B** Volcano plot showing TIL-specific genes from 813 DEGs. **C** Venn diagram showing overlapping of ICT-outcome-associated and TIL-specific genes

In scRNA-seq analysis, we selected the DEGs with an absolute value of log2FC > 0.5 from CD103^+^LAG3^+^CD8^+^ T-cell and CD103^+^LAG3^+^ B-cell subsets when compared with other T or B-cell subsets. Afterward, 813 DEGs from both CD103^+^LAG3^+^CD8^+^ T-cell and CD103^+^LAG3^+^ B-cell signatures (Additional file 1: Table S4 and S5) were obtained. Based on bulk RNA-sequencing data of patients with ICT outcomes (GSE177043), 131 out of 813 DEGs were identified to be significantly associated with ICT outcomes (Fig. 3A and Additional file 1: Table S6). Meanwhile, 813 DEGs were screened based on their expression in non-lymphocyte populations, and 58 of them were found to be uniquely expressed in TILs (Fig. 3B and Additional file 1: Table S7). Among the 131 and 58 genes, 31 overlapping genes were identified as TIL-specific genes associated with ICT outcomes (Fig. 3C).

### CLTRP score for breast cancer prognosis prediction

**Fig. 4:**
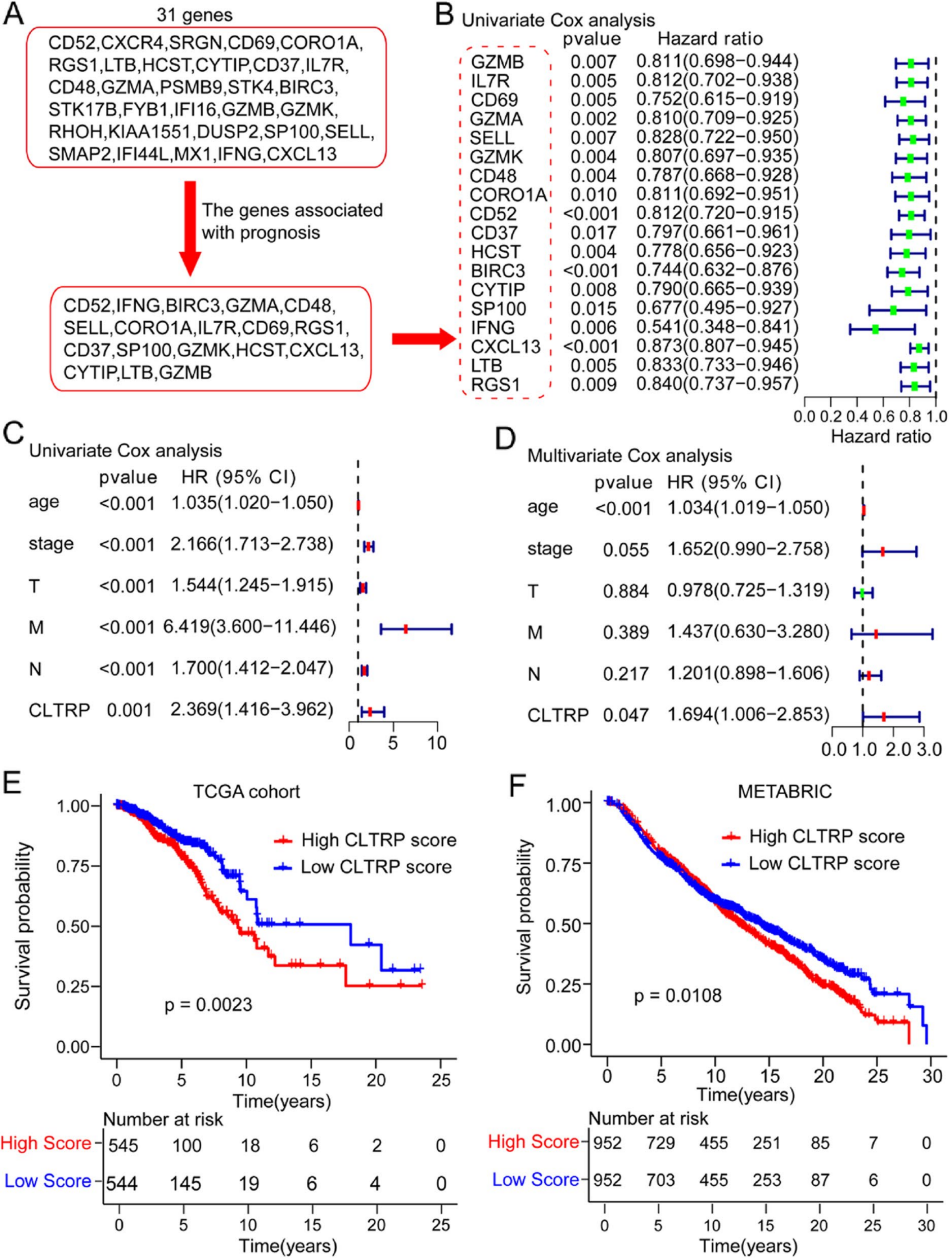
Development and validation of the CLTRP score in breast cancer cohorts. **A** Screening workflow of 31 genes from Fig. 3C. **B** Univariate Cox analysis of 18 candidate genes associated with prognosis. **C** Forest plot of univariate Cox analysis for clinicopathological factors and the CLTRP score. **D** Forest plot of multivariate Cox analysis of the factors significant in the univariate Cox analysis (*p* < 0.05). **E**, **F** Kaplan–Meier survival curve of overall survival between the high and low CLTRP score groups in the TCGA cohort and METABRIC cohort

Among 31 genes, 18 genes associated with prognosis were found (Fig. 4A) and screened using univariate Cox regression analysis (Fig. 4B). LASSO Cox regression was used to further analyse the 18 candidate genes and screen out the most appropriate gene group (CXCL13 and BIRC3) to construct a CD103^+^LAG3^+^ TIL-related risk score prognostic model (CLTRP) (Additional file 2: Fig. S3A, B). The formula is as follows: CLTRP score = -0.0877 × *CXCL13* expression – 0.2125 × *BIRC3* expression. The distribution plot of the risk score revealed that the survival times of breast cancer patients increased with a decrease in CLTRP score (Additional file 2: Fig. S3C). Univariate Cox regression analysis showed that age, stage, and CLTRP score were significantly associated with the prognosis of breast cancer (Fig. 4C). Multivariate Cox regression analysis confirmed that the CLTRP score was an independent prognostic factor after adjusting for other clinicopathologic factors (Fig. 4D).

Taking the median CLTRP as the cut-off value, CLTRP-low patients had better overall survival than CLTRP-high patients in the TCGA cohort (Fig. 4E). Then, the role of the CLTRP was further validated in the METABRIC cohort. As shown in Fig. 4F, the patients in the CLTRP-low subgroup had a significantly better prognosis than those in the CLTRP-high subgroup, consistent with the results of the TCGA dataset. In addition, based on the nomogram developed in this study (Additional file 2: Fig. S3D), the score of breast cancer patients can be calculated to predict the 1-year, 3-year and 8-year overall survival for individuals. The ability of the CLTRP score to predict 1-, 3-, and 8-year survival was indicated by AUC values of 0.616, 0.623, and 0.659, respectively (Additional file 2: Fig. S3E).

### The molecular characteristics of distinct CLTRP subgroups

**Fig. 5:**
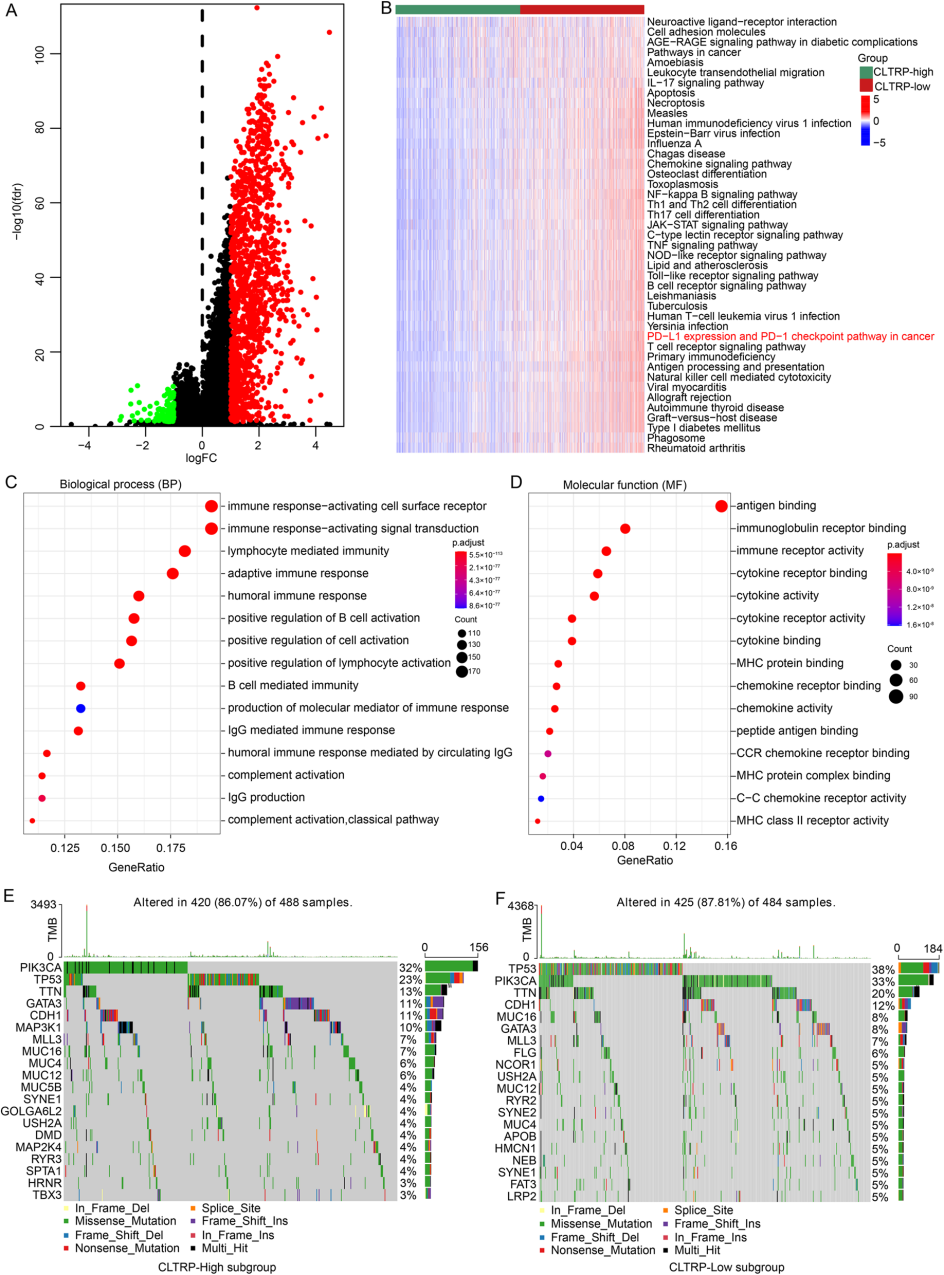
Molecular characteristics of different CLTRP subgroups. **A** Volcano plot showing differentially expressed genes (DEGs) between the high and low CLTRP subgroups. **B** GSVA of biological pathways between two different CLTRP subgroups. **C**, **D** GO enrichment analyses of DEGs among two subtypes. **E**, **F** The waterfall plot showing significantly mutated genes in the breast cancer samples of different CLTRP subgroups

DEGs were identified between the two CLTRP subgroups and displayed in a volcano plot (Fig. 5A), and the DEGs were selected for further KEGG and GO analyses. GSVA showed that the CLTRP-low subgroup was significantly enriched in many immune-related pathways, such as IL-17 signalling pathway, Th1 and Th2 cell differentiation, the B-cell signalling pathway, the T-cell signalling pathway, and natural killer cell-mediated cytotoxicity (Fig. 5B). We noticed that PD-L1 expression and the PD-1 checkpoint pathway were significantly enriched in the CLTRP-low subgroup (Fig. 5B), implying that a low CLTRP score was associated with a greater benefit from immunotherapy. In addition, we found that in the GO analysis, these DEGs were enriched in immune-related terms, such as immune response-activating signal transduction, lymphocyte mediated immunity, adaptive immune response, humoral immune response, positive regulation of B cell activation, positive regulation of lymphocyte activation, B-cell mediated immunity, and complement activation in the biological process (BP) category (Fig. 5C and Additional file 2: Fig. S4A); antigen binding, immunoglobulin receptor binding, and immune receptor activity in the molecular function (MF) category (Fig. 5D and Additional file 2: Fig. S4B); and immunoglobulin complex, T-cell receptor complex, and plasma membrane signalling receptor complex in the cellular component (CC) category (Additional file 2: Fig. S4C, D). These results preliminarily showed the functional differences between the two CLTRP subgroups of breast cancer patients, and these differential terms may be promising targets for intervention to improve prognosis.

To gain further biological insight into the immunological nature of the CLTRP subgroups, gene mutations were compared in breast cancer patients with high and low scores. We found that missense mutations were the most common mutation type, followed by nonsense and frameshift deletions (Fig. 5E, F). We then identified the top 20 genes with the highest mutation rates in the CLTRP subgroups. The mutation rates of TP53, PIK3CA, TTN, CDH1, MLL3, MUC16, MUC12, and GATA3 were higher than 5% in both CLTRP subgroups (Fig. 5E, F). Mutations in GATA3 and MAP3K1 were more common in the CLTRP-high subgroup (Fig. 5E, F), while mutations in TP53 and TTN were more common in the CLTRP-low subgroup (Fig. 5E, F).

### The immune characteristics of distinct CLTRP subgroups

**Fig. 6:**
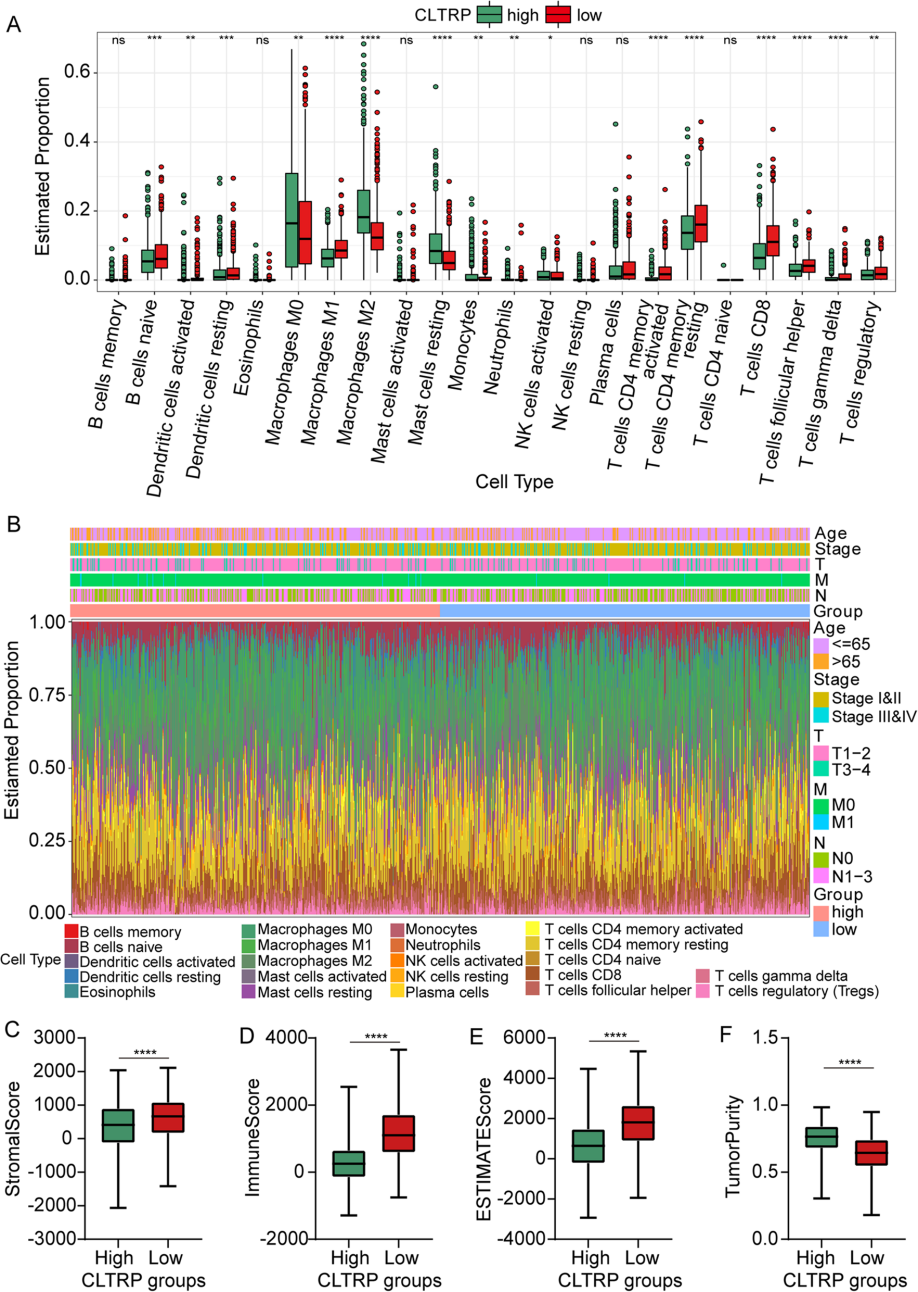
The landscape of immune cell infiltration and immune characteristics of different CLTRP subgroups. **A** The landscape of immune cell infiltration between different CLTRP subgroups. **B** CLTRP grouping and proportions of immune cells in breast cancer patients in the TCGA cohort. Age and tumour stage are shown as patient annotations. **C-F** Stromal score, immune score, ESTIMATE score, and tumour purity between the two CLTRP subgroups. *p < 0.05, **p < 0.01, ***p < 0.001, ****p < 0.0001, measured by unpaired t test

To explore the composition of immune cells in different CLTRP subgroups, we used the CIBERSORT algorithm to elucidate the relationship between the two subgroups and 22 human immune cell subsets. We found that M0 and M2 macrophages were more abundant in the CLTRP-high subgroup, while CD4 T cells, CD8 T cells, naïve B cells, and dendritic cells were more abundant in the CLTRP-low subgroup (Fig. 6A). Characteristics related to the immune landscape, including the clinicopathological characteristics of different CLTRP subgroups, are displayed in Fig. 6B. We also assessed the tumour microenvironment (TME) score (immune score, ESTIMATE score and stromal score) of the different subgroups using the ESTIMATE package and noted that the stromal score, immune score and ESTIMATE score were significantly higher in the CLTRP-low subgroup (Fig. 6C-E), while tumour purity was significantly higher in the CLTRP-high subgroup (Fig. 6F). These results suggested that the immune infiltration of breast cancer patients was associated with the CLTRP score, suggesting that patients with a low CLTRP score may benefit more from immunotherapy.

### The prediction of chemotherapeutic benefit based on the CLTRP value

**Fig. 7:**
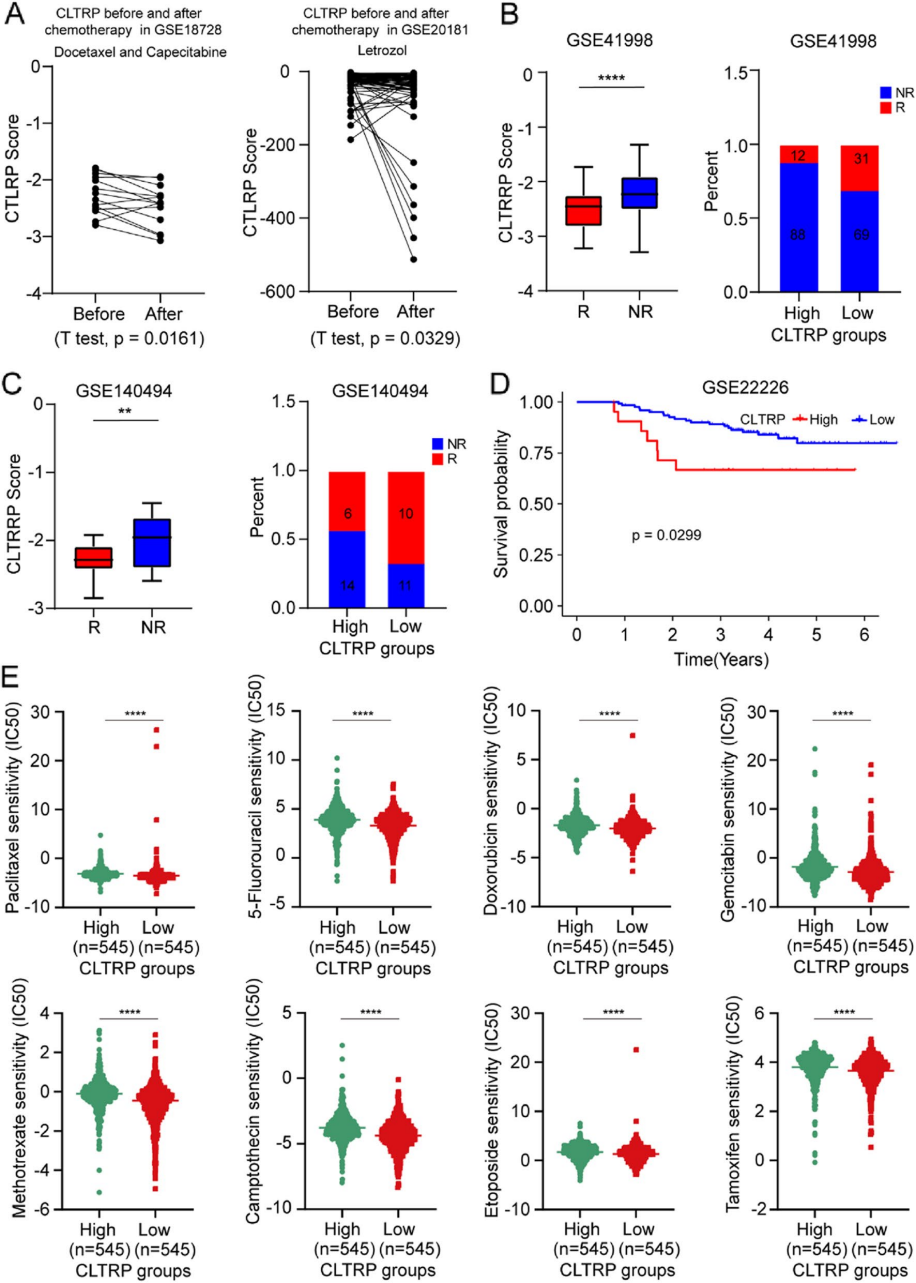
The ability of the CLTRP score to predict chemotherapeutic benefit. **A** Pairwise comparison of the CLTRP score in patients before and after chemotherapy. **B**, **C** Boxplot exhibiting the distribution of the CLTRP score for breast cancer patients with different chemotherapy responses. Right panel: Bar graph displaying the numbers of clinical responses to chemotherapy in the high and low CLTRP subgroups. **D** The CLTRP-low subgroup receiving chemotherapy is associated with improved prognosis. **E** The IC50 values of chemotherapeutic drugs in the high and low CLTRP subgroups. **p < 0.01, ***p < 0.001, ****p < 0.0001, measured by unpaired t test

Chemotherapy is one of the first-line treatments for breast cancer. To explore the relationship between CLTRP score and chemotherapy, the CLTRP value in breast cancer patients who received adjuvant chemotherapy was investigated in two cohorts: GSE18728 and GSE20181. These two breast cancer cohorts included gene expression data of patients before and after adjuvant chemotherapy. Pairwise comparisons for the CLTRP values in these two cohorts showed statistically significant differences between patients before and after adjuvant chemotherapy (Fig. 7A). Breast cancer patient samples after adjuvant chemotherapy displayed a statistically significant reduction in CLTRP values when compared with the paired prechemotherapy samples (Fig. 7A). According to the patient response to neoadjuvant chemotherapy, the breast cancer patients in the GSE41998 and GSE14094 cohorts were divided into two groups: the nonresponse (NR) group and the response (R) group. Figure 7B, C demonstrates that in the GSE41998 and GSE14094 datasets, the CLTRP values of the breast cancer patients in the R group were significantly lower than those of the breast cancer patients in the NR group. We also verified the effectiveness of the CLTRP value in predicting the response to chemotherapy in breast cancer patients. The distributions of NR and R across the different CLTRP subtypes were assessed. We found that breast cancer patients in the low CLTRP subgroup had a better response to chemotherapy than breast cancer patients in the high CLTRP subgroup (Fig. 7B, C). Furthermore, we evaluated the relationship between CLTRP subgroups and prognosis in breast cancer patients receiving chemotherapy. The results showed that breast cancer patients with a low CLTRP score had an improved prognosis (Fig. 7D).

We next selected first-line chemotherapy drugs currently used for the treatment of breast cancer to evaluate the sensitivity of distinct CLTRP subgroups to these drugs. The IC50 values of eight common chemotherapy drugs for each breast cancer patient were calculated by the pRRophetic package. By comparing the difference in IC50 values between the two subgroups, we found that the CLTRP-low subgroup was sensitive to all drugs, including paclitaxel, 5-fluorouracil, doxorubicin, gemcitabine, methotrexate, camptothecin, etoposide, and tamoxifen (Fig. 7E).

### The benefit of immune checkpoint therapy in different CLTRP subgroups

**Fig. 8:**
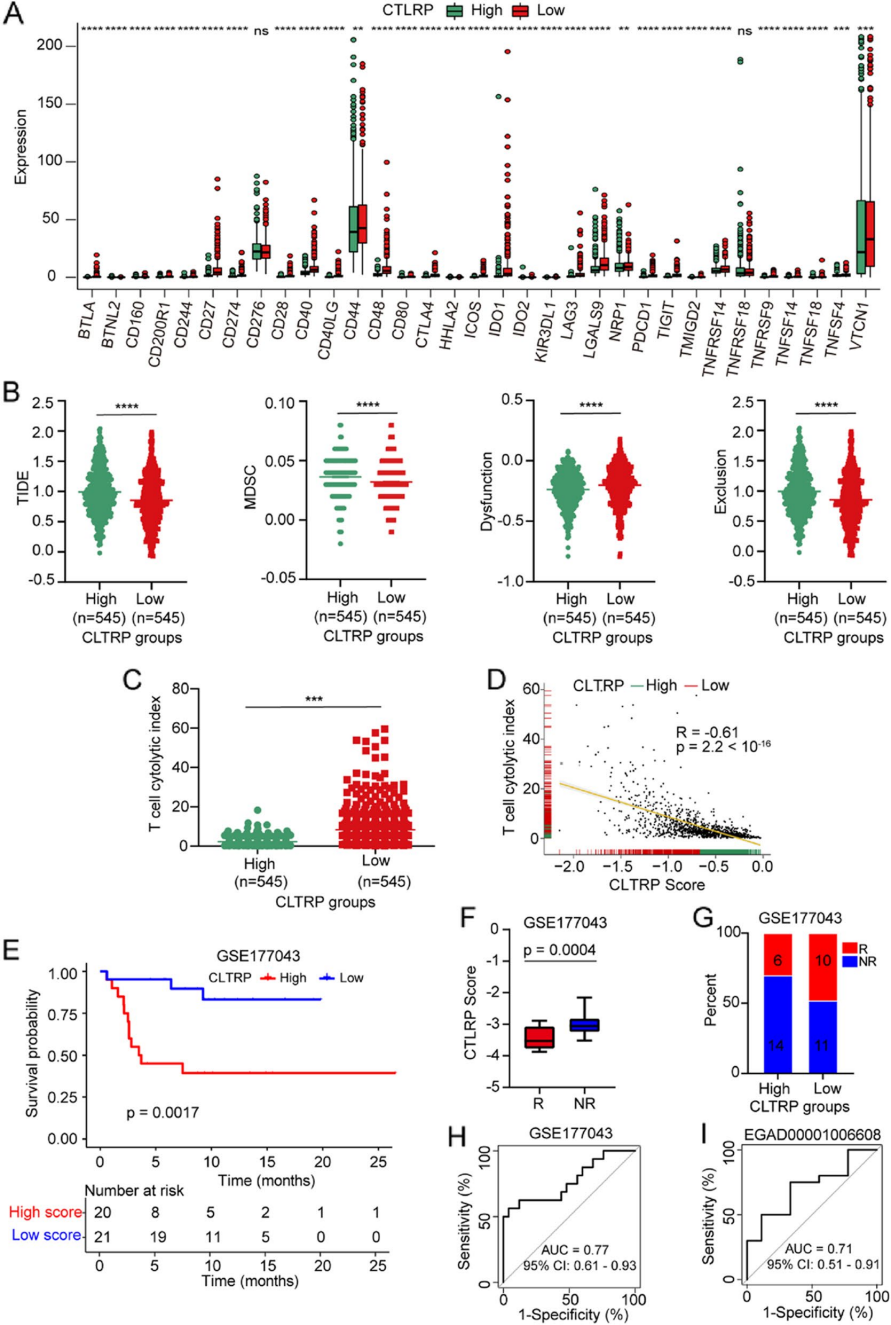
The prognostic value of the CLTRP score in breast cancer patients with anti-PD-1/PD-L1 therapy. **A** The expression of immune checkpoints in the high and low CLTRP subgroups. **B** TIDE, MDSC infiltration, and dysfunction score in the high and low CLTRP subgroups. **C** The T-cell cytolytic index in the high and low CLTRP subgroups. **D** Correlation curves between the CLTRP score and T-cell cytolytic index. **E** Kaplan‒Meier curves of overall survival according to CLTRP subgroups in the GSE177043 cohort. **F** Boxplot showing the distribution of the CLTRP score between responders and nonresponders. **G** Bar graph displaying the numbers of patients with clinical responses to anti-PD-1 immunotherapy in the high and low CLTRP subgroups. **H** and **I** CLTRP accurately predicted immunotherapy outcomes in the GSE177043 (**H**) and EGAD00001006608 (**I**) cohorts. *p < 0.05, **p < 0.01, ***p < 0.001, ****p < 0.0001, measured by unpaired t test

As shown in Fig. 8A, differential expression of 33 immune checkpoints, including PD-1, CTLA4, LAG3, and TIGIT, was observed between two distinct CLTRP subgroups. The patients in the CLTRP-low subgroup highly expressed PD-1 (PDCD1), LAG3, and CTLA4 (Fig. 8A). This suggests that patients in the CLTRP-low subgroup are more likely to benefit from ICT.

We then used tumour immune dysfunction and exclusion (TIDE) score to assess the potential clinical efficacy of immunotherapy [37] in different CLTRP subgroups. A lower TIDE score represented a higher potential for immune surveillance, which suggested that the patients were more likely to benefit from ICT. In our results, the CLTRP-low subgroup had a lower TIDE score than the CLTRP-high subgroup, implying that CLTRP-low patients could benefit more from ICT than CLTRP-high patients (Fig. 8B). In addition, we found that the CLTRP-low subgroup had a lower myeloid-derived suppressor cell (MDSC) score and a higher T-cell dysfunction score between the two subgroups (Fig. 8B). We also analysed the T-cell cytolytic index [34] in different CLTRP subgroups. We found that the CLTRP-low subgroup had a higher T-cell cytolytic index than the CLTRP-high subgroup (Fig. 8C). In addition, we observed a negative correlation between the T-cell cytolytic index and CLTRP score in the TCGA cohort (Fig. 8D). These results showed that a low CLTRP score was associated with an immunocompetent microenvironment and thus contributed to increased efficacy of immunotherapy.

Currently, most patients with breast cancer do not respond to immunotherapy, and there are no validated biomarkers for predicting the response [38]. In this study, we further explored whether the CLTRP value could predict the outcomes of immunotherapy for breast cancer patients. The breast cancer cohorts (GSE177043 and EGAD00001006608) receiving anti-PD-1/PD-L1 therapy were enrolled and divided into high and low CLTRP subgroups. We assessed the prognostic value of the CLTRP score in the GSE177043 cohort treated with anti-PD-L1 therapy (the EGAD00001006608 cohort lacked survival information). Kaplan–Meier curves showed a low CLTRP score was associated with improved overall survival in breast cancer patients (Fig. 8E). The boxplots further showed that responders had lower CLTRP scores than nonresponders (Fig. 8F). A low CLTRP score was associated with a better response rate (Fig. 8G). We also evaluated the performance of the CLTRP score in predicting immunotherapy outcomes of breast cancer patients using these two cohorts. For the GSE177043 cohort, the predictive performance of the CLTRP score was indicated by an AUC = 0.77 (95% CI, 61–93%) (Fig. 8H). For the EGAD00001006608 cohort, the CLTRP score also accurately predicted ICT outcomes with an AUC = 0.71 (95% CI, 51–91%) (Fig. 8I). These results suggest that breast cancer patients with a low CLTRP score are more likely to benefit from ICT. Moreover, our present study suggests that CLTRP is a promising marker for predicting the immunotherapy response.

## Discussion

As one of the most powerful techniques for analysing the complexity of solid tumours [20], scRNA-seq analysis of primary tumours has enabled the discovery of novel, clinically relevant cell subsets defined by a unique signature of gene expression [39–41]. In primary human tumours, transcriptome analysis based on scRNA-seq not only reveals the heterogeneity of T cells and B cells but also has begun to clarify dynamic relationships between T-cell subpopulations [42–44]. These strategies can be used to assess the conditional relationships between T-cell subsets or B-cell subsets and the clinical features of cancer. For example, Peter and colleagues used scRNA-seq analysis to uncover that breast cancer tissues contain large quantities of TILs [30]. Their work led to the discovery of an intratumoural CD8^+^ tissue-resident memory T (TRM) cell subset associated with improved prognosis [30]. In the present work, based on integrated analysis of scRNA and bulk RNA sequencing data and machine learning algorithms, we identified CD8^+^ T-cell and B-cell subsets with high expression of CD103 and LAG3 and developed a CLTRP scoring system based on signature genes from the above subsets as a predictive model for immunotherapy outcomes of breast cancer patients.

CD103, a marker expressed on CD8^+^ T cells, triggers lytic granule polarization and release at contact areas, leading to the killing of tumour cells [10, 45]. Thus, CD103 is essential for antitumour cytotoxic T-cell activity. Moreover, CD103 binds to its ligand E-cadherin on epithelial tumour cells, leading to the retention of antigen-specific lymphocytes within epithelial tumours [46]. Therefore, CD103 functions as a marker of TRM cells. CD103-positive TILs have been reported to be associated with improved prognosis in patients with triple-negative breast cancer [47]. In this study, we found that CD103 alone could not predict breast cancer survival. This may be attributed to tumour heterogeneity. LAG3, an immune checkpoint molecule, is used to mark exhausted T cells in an increasing number of studies [14–19].

In the present work, we identified two tumour-infiltrating lymphocyte subsets with high expression of CD103 and LAG3, including a CD8^+^ T-cell and a B-cell subset. These two subsets were named CD103^+^LAG3^+^ lymphocytes for convenience of description. The CD103^+^LAG3^+^CD8^+^ T-cell subset is similar to TRM cells, and this subset highly expressed cytotoxic genes, such as PRF1 and GZMB. This molecular phenotype suggests a relationship between the CD103^+^LAG3^+^CD8^+^ T-cell subset and improved prognosis of breast cancer patients. In addition, effector memory B cells have recently emerged as crucial targets for immunotherapy that could be clinically beneficial for patients with solid tumours [48–50]. Furthermore, our present work showed that the CD103^+^LAG3^+^ B-cell subset highly expressed marker genes of effector memory B cells, suggesting that this subset is associated with improved prognosis. Based on TCGA datasets, we screened two genes (CXCL13 and BIRC3) from the above two subset signatures by a machine learning algorithm called LASSO Cox regression and developed a CLTRP scoring system. In melanoma, lung cancer, and colorectal cancers, CXCL13, along with CCR5, has been identified as a T-cell-intrinsic marker of ICT sensitivity [51]. In high-grade serous ovarian cancer, CXCL13 increases infiltration of TILs and is helpful to enhance efficacy of ICT [52]. In present study, integrated analysis of scRNA-sequencing data derived from primary breast cancer and bulk RNA-sequencing data from patients receiving ICT identified CXCL13 and BIRC3 as TIL-related markers of ICT sensitivity in breast cancer.

In this study, there are some limitations. First, all prognostic analyses were performed solely on data from public databases. Therefore, larger preclinical studies and retrospective clinical trial analyses are required to confirm our findings. Second, given that breast cancer cohorts in our study were from different public datasets, intratumor or interpatient heterogeneity was unavoidable. It has been reported that tumour heterogeneity is closely associated with the efficacy of immunotherapy or chemotherapy. Despite these limitations, the present study suggests that CLTRP is a promising biomarker for determining prognosis, chemotherapeutic drug sensitivity and immune benefit from ICT in breast cancer patients and may be helpful for clinical decision-making in breast cancer patients.

## Conclusions

In the present study, we identified two TIL subsets with high expression of CD103 and LAG3 via scRNA-seq analysis. Based on The Cancer Genome Atlas (TCGA) dataset, we constructed a CD103^+^LAG3^+^ TIL-related risk score prognostic model (CLTRP) for patients with breast cancer. This CLTRP signature could accurately predict the prognosis, drug sensitivity, molecular and immune characteristics, chemotherapy benefit and immunotherapy outcomes of breast cancer patients. CLTRP could therefore serve as a predictor of both prognosis and treatment response for breast cancer.

## Supplementary Information

**Additional file 1: Table S1.** The website summary of all R packages used in this study. **Table S2.** Significant signature genes of all T cell subsets. **Table S3.** Significant signature genes of all B cell subsets. **Table S4.** Significant signature genes of CD103+LAG3+CD8+ T cell subset. **Table S5.** Significant signature genes of CD103+LAG3+ B cell subset. **Table S6.** The statistically significant genes associated with ICT outcomes. **Table S7.** TILs-specific genes.

**Additional file 2: Fig.** **S1.** tSNE plot of immune cells. **Fig. S2.** Characterization of tumour-infiltrating lymphocytes. **Fig. S3.** Nomogram developed for predicting the probability of 1-, 3- and 8-year overall survival in the training cohort. **Fig. S4.** GO analysis of differential expressed genes.

## Data Availability

All data used in this study are publicly available as described in the Method section. The web links or unique identifiers for public cohorts/datasets are described in the paper.

## Abbreviations

TILs: Tumour-infiltrating lymphocytes
CLTRP: CD103^+^LAG3^+^ tumour-infiltrating lymphocyte-related risk score prognostic model
LASSO: Least absolute shrinkage and selection operator
ICT: Immune checkpoint therapy
TRM: Tissue-resident memory T cell
LAG3: Lymphocyte activation gene 3
scRNA-seq: Single-cell RNA sequencing
TCGA: The Cancer Genome Atlas
METABRIC: Molecular Taxonomy of Breast Cancer International Consortium
ROC: Receiver operating characteristic
AUC: Area under the curve
TIDE: Tumour Immune Dysfunction and Exclusion
DEGs: Differentially expressed genes
GO: Gene ontology
KEGG: Kyoto Encyclopedia of Genes and Genomes
GSVA: Gene set variation analysis
MSigDB: Molecular Signatures Database
MAF: Mutation annotation format
IC50: Half-maximal inhibitory concentration
t-SNE: T-distributed stochastic neighbour embedding
GZMB: Granzyme B
PRF1: Perforin
BP: Biological process
MF: Molecular function
CC: Cellular component
TME: Tumour microenvironment
HR: Hazard ratio
