# Supplementary material for: The expression profiles of signature genes from CD103+LAG3+ tumour-infiltrating lymphocyte subsets predict breast cancer survival
Source: BMC Med. 2023 Jul 24;21:268. doi: 10.1186/s12916-023-02960-1 (PMC10367329; doi:10.1186/s12916-023-02960-1)
Supplement: Supplementary file 2 — Additional file 2: Fig. S1. tSNE plot of immune cells. Fig. S2. Characterization of tumour-infiltrating lymphocytes. Fig. S3. Nomogram developed for predicting the probability of 1-, 3- and 8-year overall survival in the training cohort. Fig. S4. GO analysis of differential expressed genes. [file 12916_2023_2960_MOESM2_ESM.docx]

Supplementary Materials for

**The expression profiles of signature genes from CD103^+^LAG3^+^ tumour-infiltrating lymphocyte subsets predict breast cancer survival**

**Zi-An Xia, Can Lu, Can Pan, Jia Li, Jun Li, Yitao Mao, Lunquan Sun*****, Jiang He***

*Corresponding author. Email: [lunquansun@csu.edu.cn](mailto:lunquansun@csu.edu.cn) and [hj2008s@csu.edu.cn](mailto:hj2008s@csu.edu.cn)

**This file includes:**

Supplementary Text

Figures S1 to S4


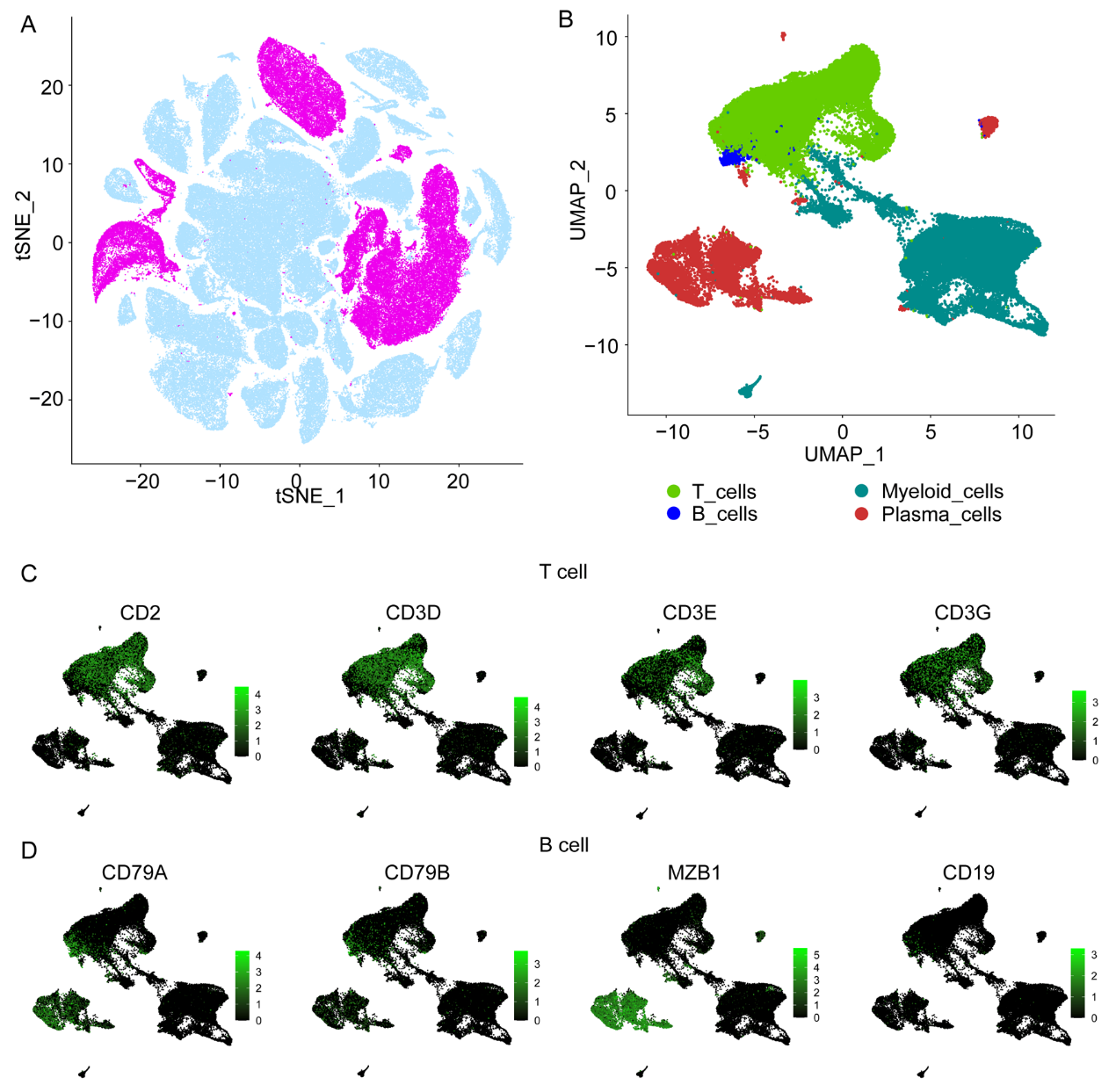


**Fig. S1** tSNE plot of immune cells. (**A**) tSNE plot exhibiting immune cells and non-immune cells. (**B**) UMAP plot displaying immune cells. (**C**) UMAP plot showing the expression of T cell marker genes. (**D**) UMAP plot showing the expression of B cell marker genes.


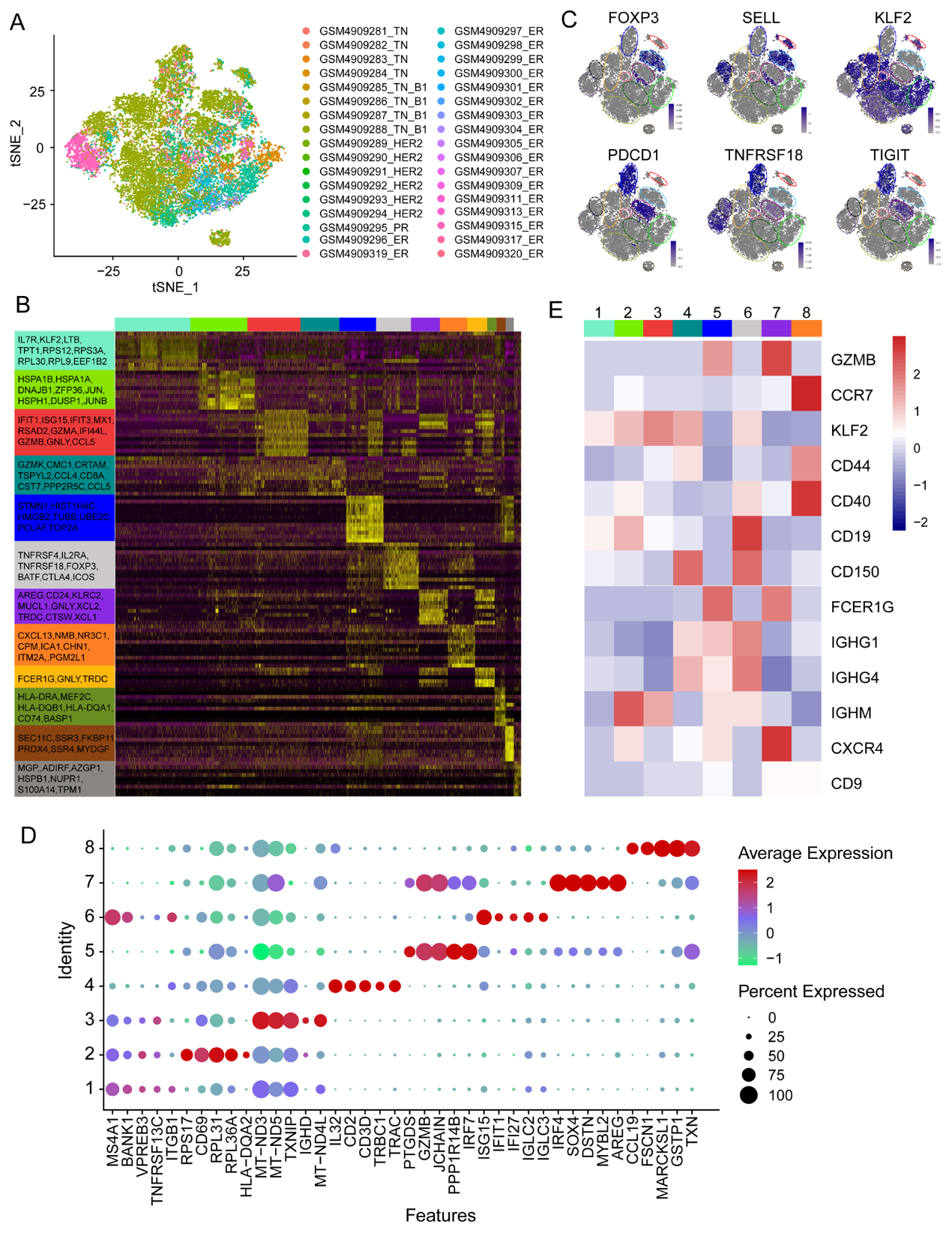


**Fig. S2** Characterization of tumor-infiltrating lymphocytes. (**A**) tSNE plot showing intra- and inter- patients heterogeneity of T cells. (**B**) The top ten genes differentially expressed for each cluster are shown on the y axis, and key genes are also shown for each cluster. (**C**) t-SNE plot exhibiting the expression of TRM marker genes and exhaustion genes. (**D**) Top five marker genes of 8 major B cell subsets identified in this profile. (**E**) Heatmap showing the expression of B cell maker genes.

**
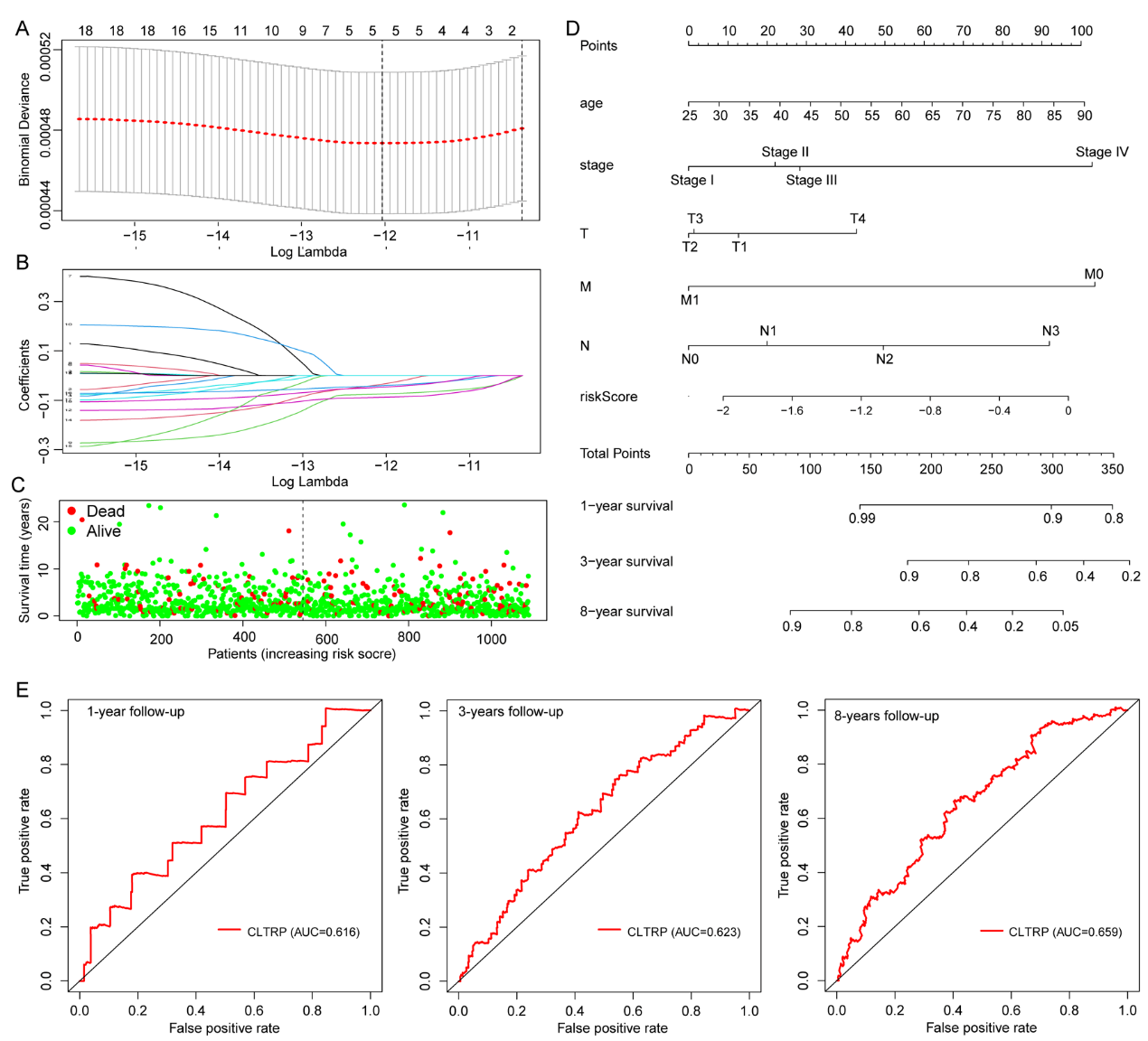
Fig. S3** Nomogram developed for predicting the probability of 1-, 3- and 8-year overall survival in the training cohort. (**A, B**) The LASSO regression analysis and partial likelihood deviance on the prognostic genes. (**C**) The ranked dot plot of the CLTRP score distribution and scatter plot of the patients’ survival status. (**D**) The nomogram was constructed in the training cohort with the CLTRP. (**E-G**) ROC curves to predict the sensitivity and specificity of 1-, 3-, and 8-years survival according to the CLTRP score.


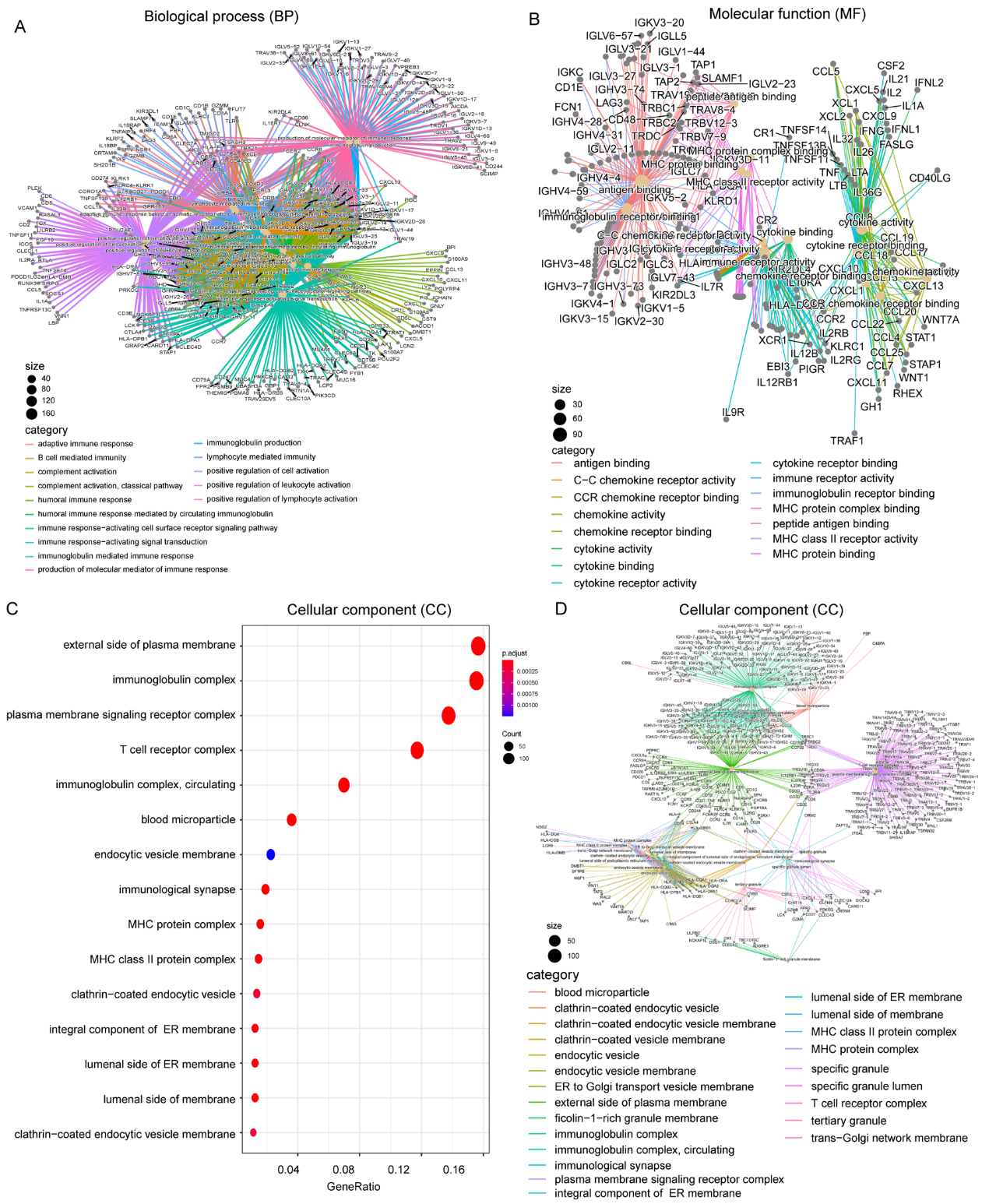


**Fig. S4** GO analysis of differential expressed genes. (**A**) The result of GO analysis in biological process. (**B**) The result of GO analysis in molecular function. (**C, D**) The result of GO analysis in cellular components.
